# Awareness on Teratogenic Effects of Isotretinoin and Compliance with Precautionary Measures among Women of Childbearing Age in Makkah Province, Saudi Arabia

**DOI:** 10.1155/2021/9966300

**Published:** 2021-04-10

**Authors:** Rawan Al-Mekhlafi, Rawabi E. Attiyah, Yara R. Haddad, Louai A. Salah

**Affiliations:** ^1^Batterjee Medical College, Obhur Prince Abdullah Al-Faisal Street, North Jeddah, Saudi Arabia; ^2^East Jeddah General Hospital, Ministry of Health, Jeddah, Saudi Arabia

## Abstract

Acne vulgaris ranks among the most common dermatologic conditions encountered during adolescence up to adulthood. For moderate to severe cases of acne, isotretinoin is indicated as it is considered the most efficacious medication against acne. However, isotretinoin use is known to have its side effects and most importantly is the drug's teratogenic potential. As a response, programs such as the Retinoid Pregnancy Prevention Program (PPP), System to Manage Accutane-Related Teratogenicity (SMART), and iPLEDGE were put into action as attempts to promote awareness on isotretinoin's teratogenicity and reduce the incidence of exposed pregnancies. Such programs are lacking in Saudi Arabia. This study aimed, therefore, to evaluate the awareness of women of childbearing age in Makkah Province, Saudi Arabia, with regards to the side effects of the medication, specifically its teratogenicity. This study also intended to assess the compliance of both doctors and patients with the recommendations and precautions associated with isotretinoin. A cross-sectional study was conducted on 766 women participants using a previously validated questionnaire. Results showed that majority of the respondents (91%) are generally aware of the side effects of isotretinoin use, particularly its teratogenicity. However, lapses have been identified with regards to the compliance of both the treating physician and the patient. Three-fourths of sexually active women did not use any form of contraception while being on isotretinoin treatment. Two-thirds of the study participants responded that they were not issued approval forms indicating their understanding of the side effects of isotretinoin and the importance of compliance to the treating physician's instructions; 11.5% claimed that their doctors did not perform any blood tests; and 67.7% claimed that no pregnancy test was performed at any time during the treatment. These findings strongly suggest a need for improvement when it comes to compliance of both doctors and patients. It is recommended that doctor-patient communication be more comprehensive and more efforts should be made to follow international guidelines in that regard.

## 1. Introduction

Acne vulgaris is a common disease involving the pilosebaceous unit [1]. According to the Global Burden of Disease (GBD) study, it affects as much as 85% of young adults aged between 12 and 25 years old [2]. It is also identified to be the most prevalent skin disease peaking around adulthood, though in some cases, it persists in older females. One noted characteristic of acne vulgaris is its effects across different domains of well-being including the physical, psychological, and social aspects [3, 4].

Isotretinoin, a vitamin A derivative, is a first-generation synthetic retinoid drug most commonly known as Accutane or Roaccutane. It has been determined to be the drug of highest efficacy against moderate up to severe cases of acne as noted by the Food and Drug Administration (FDA) [5]. Despite the established favourable reputation of isotretinoin in acne therapy, there have been reports on its adverse effects including dry skin and mucous membranes, elevated lipid profile, and recurrent muscle aches. It is also known to be highly teratogenic [6, 7]. The effects can range from fetal cardiovascular defects, central nervous system birth defects (hydrocephalus), craniofacial defects (microtia), up to spontaneous abortions. For these reasons, isotretinoin is contraindicated for use during pregnancy and has since carried a category X pregnancy classification [8]. A number of risk preventive programs aimed at pregnancy avoidance and prevention in women of childbearing age have been implemented following the introduction of this medication. In 1988, the Retinoid Pregnancy Prevention Program (PPP) was implemented by a pharmaceutical company called Roche along with the US FDA. The program included explanation of the risks associated with teratogenicity of isotretinoin, as well as collection of written agreements from women of childbearing age to follow two contraceptive methods spontaneously while on medication [9–11]. The European Commission also decided to implement the PPP program to all isotretinoin products as early as 2003. However, despite these efforts, records of exposed pregnancies still persisted since, and the recommendations seem to have not been strictly followed by health care professionals and patients. A Dutch study reported 143 cases of exposed pregnancies in 22 different countries of the European Union between 2003 and 2004. In France, about 147 exposed pregnancies have been reported from 2003 until 2006, while Swissmedic recorded approximately 4–9 exposed pregnancies annually, with 6% of these leading to birth defects [10]. A 2008 Saudi Arabian study recorded 7 pregnancies out of 792 women receiving isotretinoin, and three (3) of these pregnancies were terminated electively [4]. In response to the failure of PPP to prevent pregnancies while on isotretinoin, a similar program called System to Manage Accutane-Related Teratogenicity (SMART) was implemented in 2002 in the United States. SMART had the same goal as PPP, albeit enforcing stricter guidelines prior to starting isotretinoin medication. This was followed by iPLEDGE program put forward by the US FDA in 2006, aiming to impose stricter regulations and documentation, and all of which proved ineffective in reducing the frequency of exposed pregnancies [9].

International studies evaluating women's awareness on the teratogenicity of isotretinoin and the compliance with the concomitant precautions are extensive. However, in the Kingdom of Saudi Arabia, published studies mainly focused on the general knowledge surrounding the side effects of isotretinoin use, but only a handful went in-depth on the teratogenic aspect of the medication [3, 11–13]. Also, other studies did not thoroughly evaluate the level of compliance among treating physicians [12]. This study aims to assess the general knowledge surrounding isotretinoin use among women of childbearing age living in the Makkah region of Saudi Arabia who are undergoing or have undergone such medication. Subjects for assessment include the awareness on the side effects of isotretinoin, its teratogenic potential, and understanding on the importance of pregnancy exclusion before treatment initiation, as well as pregnancy prevention during treatment. Furthermore, this study aims to determine their compliance level with pregnancy prevention efforts during the treatment course which includes effective contraception methods. Results of this study are expected to contribute towards understanding and monitoring the current situation in the region, and ultimately in implementing more effective efforts in reducing isotretinoin-related exposed pregnancies.

## 2. Materials and Methods

This study utilized a cross-sectional approach to determine the awareness on the teratogenic effects of isotretinoin, as well as the compliance to precautionary measures among women of childbearing age (12–52) in Makkah Province, Saudi Arabia, who were undergoing or have undergone treatment with isotretinoin. This research was conducted from February 2019 to May 2019, and nonresidents of Makkah province were excluded from the study. An 8-item online questionnaire that was developed by the authors and previously validated was deployed through social media platforms, e.g., Snapchat, Instagram, Twitter, WhatsApp, and Telegram, capitalizing on their accessibility and convenience [14]. The questionnaire also has both Arabic and English versions, subject to the preference of the study participant. Self-reported data mainly consisted of sociodemographic information, response to questions regarding awareness and compliance, and patient perspective on the treating physician's practice. This study was approved by both the Research Ethical Committee of Batterjee Medical College and the Institutional Review Board in Jeddah Directorate of Health Affairs, Ministry of Health, Saudi Arabia.

Statistical analyses of collected data were performed using IBM SPSS version 23 (IBM Corp., Armonk, N.Y., USA). The chi-square test was used to evaluate the relationship between categorical variables. In comparing group means, an independent *t*-test was used for two group means while one-way ANOVA was used in comparing more than two groups. The post hoc test used was the least significant difference (LSD). These tests rest on the assumption of a normal distribution. In cases of a non-normal distribution, Welch's *t*-test for two group means was used. The criteria to reject the null hypothesis were set to be the conventional *p* value of *p* < 0.05.

## 3. Results

### 3.1. Demographics

Seven hundred sixty-six (766) women of childbearing age undergoing isotretinoin treatment agreed to participate in the study.  Table 1 shows the sociodemographic characteristics of the study participants.

### 3.2. Isotretinoin Treatment

Table 2 shows the summary of the nature of isotretinoin treatment received by the study participants.

### 3.3. Treating Physician's Practice

The study participants were asked whether the treating physician asked them to sign an approval form indicating that the patient understood and received the information on the risks and precautions associated with isotretinoin. About 68% of the study participants claimed that they were not issued a consent form, and 28% responded that their treating physician issued a consent form, while 4% did not follow up with a doctor.

In the proportion of women who were sexually active (*n* = 96), 22 reported that their doctor conducted a pregnancy test only prior to isotretinoin treatment; two responded that a pregnancy test was conducted before and during treatment; and another two participants said that a pregnancy test was performed before, during, and after treatment. 65 women claimed that a pregnancy test was never conducted in their case.

**Figure 1:**
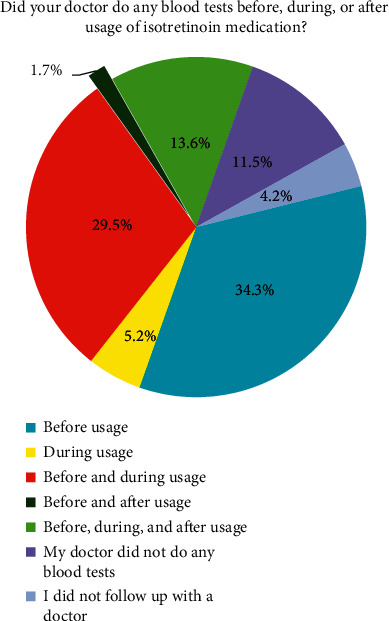
Responses of study participants whether their doctors performed blood tests before, during, or after isotretinoin treatment.

The study participants were also asked whether their treating physician performed blood tests before, during, or after isotretinoin treatment.  Figure 1 summarizes their responses.

### 3.4. Women's Practice and Awareness

Approximately, 95% of the study participants were able to follow up, at least once, with their treating physician throughout the course of treatment. The remaining 5% responded that they did not do any follow-up at all.

**Figure 2:**
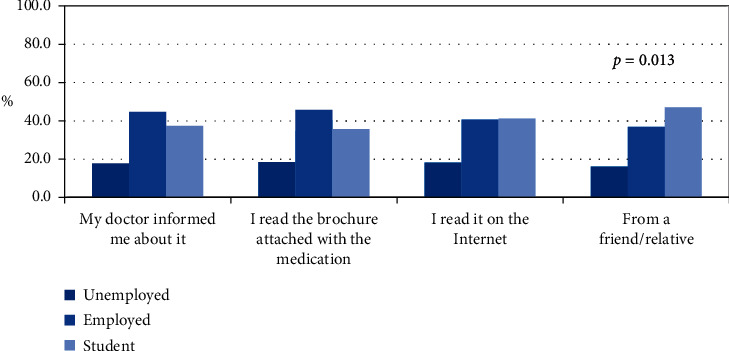
Relationship of employment status with source of information on side effects.

In terms of knowledge on side effects of isotretinoin, 91% of the study participants claimed to be informed of the possible side effects, of which 71% cited their doctors as their primary source of information. The remaining 29% mentioned other sources of information such as the Internet, brochures attached to the medication, and/or friends or relatives. In relation to employment status, knowledge on side effects derived from friends/relatives showed a *p* value of 0.013, as shown in Figure 2.

The most common side effects reported include dryness of the skin, lips, and eyes, accounting for 87.6% of the study participants. Nosebleeds were experienced by 15.4% of women under study while the rise of blood cholesterol level has been reported in 12.9%.

Ninety-six (96) study participants were identified to be sexually active during the treatment, and half of whom are unmarried. Two-thirds of these women did not inform their doctors about being sexually active during treatment, and three-fourths did not use any method of contraception. In the proportion of women who used contraceptives, only 28% responded that they are strictly committed and are always adherent to using them. Out of the 96 sexually active women, three participants reported pregnancy during treatment, and two of which underwent normal delivery while the other one had a miscarriage.

Lastly, results showed that majority of women (approx. 93%) both from the sexually active and sexually inactive group were knowledgeable in the teratogenic effects of isotretinoin and pregnancy avoidance during and one month after treatment.

## 4. Discussion

This study aims to investigate the awareness of women of childbearing age in the province of Makkah, Saudi Arabia, regarding the teratogenicity of isotretinoin and patients' compliance with the precautionary measures associated with the medication. There have been similar studies conducted in different regions of Saudi Arabia including Western region, Al-Madinah, Riyadh, Qassim, and Al Ahsa, albeit focusing on the general understanding of isotretinoin usage and the concomitant compliance [5, 12, 15–17]. However, this study delved deeper on the teratogenic aspect of isotretinoin and its relation to pregnancy, highlighting the importance of safety of the childbearing woman and the fetus. Two primary aspects were assessed by the previously validated questionnaire: women's practice and awareness, and their treating physician's practice [14].

### 4.1. Demographics and Treatment

Results from the sociodemographic characteristics of the study participants revealed that the majority of the respondents are aged between 19 and 25 years old. In terms of acquisition of the medication, it can be noticed that a proportion of women were able to obtain isotretinoin from sources such as the Internet, friends/relatives, or pharmacies even without a prescription from a doctor. On a similar note, a small proportion of the respondents used isotretinoin for nonacne related reasons. Isotretinoin misuse can vary; it can manifest in improper dosage and duration of treatment (i.e., very low/high dosage and very short/long duration of treatment), which are not in accordance to clinical guidelines [18, 19]. Improper usage of isotretinoin medication can affect the efficacy of the treatment and can contribute to possible relapses in the future. Blasiak et al. (2013) noted that lower doses of isotretinoin (<220 mg) have been associated with higher rates of relapse, as compared to the groups prescribed with higher doses (>220 mg) [20]. In contrary, a 2020 Saudi study found a high relapse rate even at increased doses (>40 mg/day for 7.15 ± 4.5 months), citing recall bias and uneven gender distribution of the participants as probable reasons for the high relapse rate encountered [21].

### 4.2. Treating Physician's Practice

Sixty-eight percent (68%) of the respondents claimed not being issued an approval form indicating their understanding of the risks and precautions associated with isotretinoin use. A significant number of women (11.5%) reported that their doctor did not perform any blood test at all throughout the duration of treatment. It is also important to note that most of the treating physicians for the sexually active group failed to comply with the recommendations in regards to conducting pregnancy tests before, during, or after treatment, as 67.7% of them did not perform any pregnancy test at any point in time. This supports the findings of a 2008 study in Riyadh, Saudi Arabia, wherein the level of compliance of attendees of a national conference on dermatology was assessed and showed that only 16% of dermatologists requested for monthly pregnancy tests [5].

### 4.3. Women's Practice and Awareness

The vast majority (91%) of study participants claimed understanding and awareness on the side effects associated with isotretinoin use, which is consistent with previous studies. Molla et al. (2020) reported that 62 of their respondents are generally aware of the side effects, while Al-Harbi (2010) noted that 77% of their study participants are knowledgeable about the medication and its uses [15, 16]. Another study conducted on Saudi women in Western Saudi Arabia revealed that around 85% of their sample population is aware of the side effects of the medication [21]. However, one point of concern is that a substantial proportion (29%) of these “knowledgeable” women in this study get their information from sources such as the Internet, brochures, and friends/relatives, and all of which pose risks of misinformation and misinterpretation. These sources are characterized by a high degree of unreliability and can ultimately lead to isotretinoin misuse. Interestingly, Figure 2 shows significant differences across employment status for those who sourced their information from friends/relatives. Employed women are more likely to derive their information from their treating physicians, as well as from brochures attached to the medication, while students are more likely to get the relevant information from the Internet and/or from friends/relatives.

Ninety-six (96) out of the 766 study participants were sexually active during the medication. Ten of these women did not follow up with a doctor at all, while 65 of them did not inform their doctor of their sexual activity. This is an indication of physicians failing to extract this relevant information, possibly due to sociocultural barriers, rendering the women hesitant to divulge such sensitive information. Another important statistic to point out is that 71 out of the 96 sexually active women did not even use any form of contraception during isotretinoin treatment. As a result, three women conceived: two of which culminated in a normal delivery, while the other one sadly resulted in a miscarriage.

### 4.4. Limitations of the Study

This study made use of a cross-sectional approach utilizing a previously validated online questionnaire to conduct a survey on the sample population [14]. Possible sources of error include recall bias, response bias especially towards sensitive information, and other inherent limitations of a cross-sectional study.

## 5. Conclusions

Results of this study showed that women of childbearing age from the Makkah province, Saudi Arabia, are generally aware of the side effects of isotretinoin, particularly its teratogenic potential. Unfortunately, there are lapses especially with regards to compliance of both patients and doctors. A significant proportion of treating physicians did not follow some of the recommendations, especially conducting pregnancy tests and blood tests throughout the treatment period. Some study participants were able to obtain isotretinoin without a doctor's prescription, and some of them also derive their information from unreliable sources (i.e., Internet and friends/relatives). Women who were sexually active during treatment did not inform their doctors about their activity, possibly due to cultural barriers and other challenges in communication. Additionally, three-fourths of sexually active women did not even use any form of contraception at any point in time while on isotretinoin; this resulted to three cases of pregnancy: two of which culminated in a normal delivery while the other one sadly ended up as a miscarriage. These data suggest that drastic measures and improvements must be sought with regards to both doctor and patient compliance. It is also recommended that the communication between the patient and the doctor be comprehensive, as well as conducive and reassuring to women.

## Figures and Tables

**Table 1:** Sociodemographic characteristics of the study participants (*N* = 766).

Study variable	N (%)
*Age group*	
12–18	19 (2.5%)
19–25	435 (56.9%)
26–32	192 (25.1%)
33–39	83 (10.8%)
40 and above	36 (4.7%)
Data unavailable	1

*Nationality*	
Saudi	690 (90.1%)
Non-saudi	76 (9.9%)

*City of residence*	
Jeddah	614 (80.2%)
Makkah	118 (15.4%)
Taif	34 (4.4%)

*Educational attainment*	
High school and less	120 (15.7%)
Bachelor's degree	584 (76.2%)
Master's and above	62 (8.1%)

*Employment status*	
Unemployed	144 (18.8%)
Employed	331 (43.2%)
Student	291 (38.0%)

*Marital status*	
Married	91 (11.9%)
Unmarried	675 (88.1%)

**Table 2:** Nature of isotretinoin treatment received by study participants (*N* = 766).

Study variable	%
*Number of treatment courses with isotretinoin*	
One	68.5
Two	20.0
More than two	11.5

*With doctor's prescription*	
Yes	95.2
No	4.8

*Year of treatment*	
2019	27.0
2014–2018	46.2
Before 2014	26.8

*Reason for isotretinoin medication*	
Acne with oily skin and visible pores	52.1
Acne only	43.5
Perceived oily skin and visible pores without acne	2.7
Unrelated to acne	1.7

*Daily maximum dose of isotretinoin*	
10–20 mg	45.2
30–40 mg	36.9
50–60 mg	4.8
≥70 mg	1.6
Unspecified	11.5

*Duration of medication*	
<5 months	33.9
5–7 months	50.0
>7 months	16.1

## Data Availability

The data used to support the findings of this study are available from the corresponding author upon request.
